# Upper Limb Ischemia Due to Arterial Thrombosis after COVID-19 Vaccination

**DOI:** 10.1155/2022/4819131

**Published:** 2022-03-09

**Authors:** Moaath M. Alsmady, Rahaf A. Al-Qaryouti, Nesrin G. Sultan, Oweis I. Khrais, Huthaifah Khrais

**Affiliations:** ^1^Department of General Surgery, University of Jordan, Amman, Jordan; ^2^Zarqa University, Zarqa, Jordan

## Abstract

This report describes a case of a 60-year-old male patient who received the first dose of the AstraZeneca vaccine and presented to the emergency department complaining of left hand pain and paresthesia. Investigations revealed upper limb ischemia; he was hospitalized for further management.

## 1. Introduction

Vaccine-induced immune thrombotic thrombocytopenia (VITT) is a complication that has been associated with the use of COVID-19 vaccines that make use of an adenovirus vector [1], namely, Vaxzevria from AstraZeneca and Covishield from Janssen [2]. VITT is characterized by thrombosis that occurs 5 to 30 days after COVID-19 vaccination [3]. The mortality rate due to VITT varies between 30 and 60%. Primarily, the patients' treatments included nonheparin anticoagulation and intravenous immune globulin [4]. COVID-19 vaccination was associated with thromboembolic events in some patients, and it is also evident that COVID-19 infection itself is commonly associated with thromboembolism [5].

Being first reported in February 2021, the syndrome is more commonly described in women, especially those younger than the age of 55 years [6]. According to an initial report released in April 2021 by the Centers for Disease Control (CDC), the incidence of VITT is 1 in 533,333 cases [7].

The clinical presentation of VITT appears to be very similar to that of heparin-induced thrombocytopenia (HIT). Symptoms of VITT include severe headache, focal neurological deficits, abdominal and extremity pain, vomiting, and in severe cases, mental status changes, coma, and death [2, 8]. The pathophysiology of HIT, where immunoglobulin G is produced and forms aggregates with the PF4-heparin complex, closely resembles that of VITT [1].

To confirm the diagnosis, thrombocytopenia, evidence of a new thrombotic event and positivity of the ELISA test for PF4 antibodies are necessary [9] based on the Brighton Collaboration, but this may miss patients who present with either thrombosis with normal platelet count or thrombocytopenia alone [8]. There were three levels of evidence depending on the confirmation of thrombosis based on imaging, surgical, laboratory findings, or clinical presentation of thrombosis or thromboembolism [8].

In the following case, we report a possible case of VITT in a patient who was admitted to Jordan University Hospital in June 2021.

## 2. Case Presentation

A 60-year-old man was in his usual state of health before presenting to the orthopedic clinic at Jordan University Hospital on May 30^th^, 2021, complaining of left hand pain. He did not report any history of trauma. There was no weakness or paresthesia of the left upper limb, nor shortness of breath or chest pain. The patient had no personal or family history of arrhythmias or thrombotic events. There were no other cardiac, respiratory, neurological, musculoskeletal, or other symptoms. He is a nonsmoker, and he does not use any regular medications. His complaint started two weeks after receiving the first dose of the AstraZeneca vaccine.

Two days later, he presented to the emergency department complaining of increasing left hand pain and paresthesia. His left index initially was pale and then became cyanotic. He only felt tired when lifting heavy objects. There were no neurological defects or other associated symptoms.

On presentation, he was alert and oriented with stable vital signs. Cardiovascular, respiratory, and neurological examinations were normal. Upper limb pulses were not palpable on his left hand. His initial INR was 1.32, and his PT and PTT levels were elevated. Other laboratory investigations are presented in Table 1. A Doppler ultrasound of his left upper limb showed a monophasic radial pulse and a triphasic ulnar pulse.

**Figure 1:**
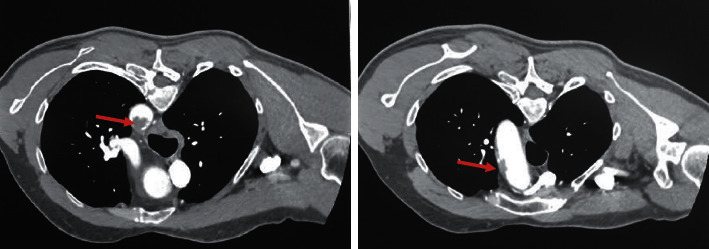
IV contrasted CT scan showing filling defect at the junction between the aortic arch and the descending aorta and an irregular mural thrombus with calcification in the aortic arch.

**Figure 2:**
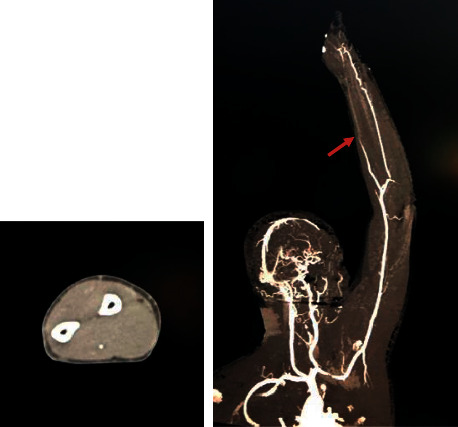
IV contrasted CT scan showing occlusion of the left radial artery at the level of the midforearm with retrograde filling at the level of the left rest.

The CT scans provided showed an irregular filling defect at the junction between the aortic arch and the descending aorta, in addition to an irregular mural thrombus with calcification involving the aortic arch (Figure 1). There was also occlusion of the left radial artery at the level of the midforearm with retrograde filling at the level of the left rest. The left subclavian, axillary, brachial, and ulnar arteries were patent (Figure 2).

**Figure 3:**
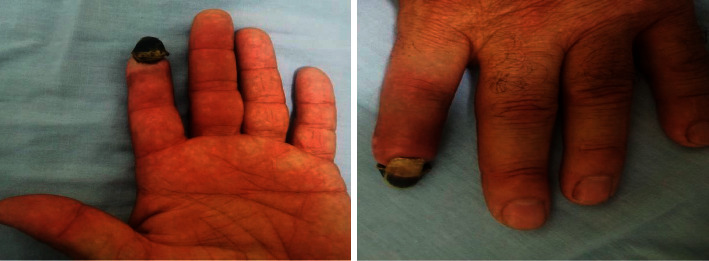
Photo of the patient's left hand showing the gangrenous distal phalanx of his left index finger.

The patient was admitted under the care of the cardiovascular team as a case of upper limb ischemia for observation and therapeutic anticoagulation. Four weeks later, our patient presented to the clinic with improvement of his symptoms and reversal of tissue ischemia except for dry gangrene in his distal phalanx of the left index, which was left for autoamputation (Figure 3).

## 3. Discussion

Thrombotic events post-COVID-19 vaccination, a type of VITT, are an evolving issue that depends on different factors such as age, gender, and geographical area [2]. These thromboembolic events are more common than other coagulopathies, such as bleeding [9]. The type of the vaccine and the number of doses also play a role, with AstraZeneca being associated with the highest incidence [2]. However, the World Health Organization released in March 2021 that the available data on AstraZeneca did not show any increased risk of clotting events despite having few cases reported on unique thromboembolism [10]. The earliest reported cases were the atypical venous thrombosis, mainly intracranial [1], which were challenging to treat [9]. Young females were mostly affected [1] and mostly presented within one to two weeks after receiving the vaccine [1, 9, 10]. The great majority of patients were previously healthy and fit [11]. So when a patient presents with headache, abdominal pain, shortness of breath, and/or limb swelling postvaccination, a platelet count and imaging for thrombosis must be carried out [12].

An epidemiological study found that thrombotic episodes were more common among older adults during the first month post-ChAdOx1 vaccine [11]. Another study found that thrombotic events were more common in females and in patients younger than 45 years [13–15].

A narrative review paper found that the association between COVID-19 vaccination and the incidence of thrombosis is low. Nonetheless, smokers and the elderly still require special attention [16]. Despite the strength of the relationship, a conclusion about the safety of COVID-19 vaccination has yet to be drawn [17].

This case is different from previous cases, as our patient was an elderly male who did not have cerebral venous thrombosis, but rather, he presented with arterial thrombosis involving the aorta and the left radial artery. A study done in England to evaluate patients who were diagnosed with VITT identified 26 patients (12%) of the sample with aortic thrombosis or limb ischemia [11].

Our patient presented with thrombosis only, and his platelet count remained within the normal range. We did not test for PF4 antibodies, as a previous study concluded that PF4 antibodies alone may not be specific as they may be positive even without symptoms. One of nine patients with thrombosis without thrombocytopenia had weakly positive antibodies but with a negative functional test [8].

Our patient was started on warfarin and INR levels were checked continuously.

To the best of our knowledge, this is the second case of VITT from the Middle East after a case of mixed arterial and venous thrombosis and thrombocytopenia that was reported in Oman [5]. Both cases were treated with nonheparin anticoagulation and showed improvement in their hematological profiles.

Much about VITT still remains unclear. It has been suggested that a vaccine-induced autoimmune response to PF4 may be plausible [18]. Another possibility is that genetic predisposition may lead to thrombotic actions; however, further studies are required on next-generation sequencing to understand this vital issue.

## 4. Conclusion

This case highlights the importance of understanding the VITT mechanism. Also, healthcare providers need to be careful of any new adverse events that may occur after the administration of COVID-19 vaccines, as patients are heterogenous and not enough evidence from the literature supports the underlying etiologies of VITT.

## Figures and Tables

**Table 1:** Laboratory results on admission.

Labs	On admission	Reference ranges
Hemoglobin	14.7	13.8–17.2 g/dL
WBCs	12.02	4.5–11 ∗103/mm3
RBCs	5.31	4.3–5.9 million/mm3
Platelets	186	150–450 ∗103/mcL
PT	17.3/13.3	11–13.5 sec
PTT	83.4/30	25–35 sec
Sodium	138	135–145 mEq/L
Potassium	3.9	3.5–5 mmol L
Urea	36	6–24 mg/dL
Creatinine	1.27	0.74–1.35 mg/dL

## Data Availability

All data generated or analyzed during this study are included within the article.
